# Pathophysiology of Early Brain Injury and Its Association with Delayed Cerebral Ischemia in Aneurysmal Subarachnoid Hemorrhage: A Review of Current Literature

**DOI:** 10.3390/jcm12031015

**Published:** 2023-01-28

**Authors:** Diana L. Alsbrook, Mario Di Napoli, Kunal Bhatia, Masoom Desai, Archana Hinduja, Clio A. Rubinos, Gelsomina Mansueto, Puneetpal Singh, Gustavo G. Domeniconi, Asad Ikram, Sara Y. Sabbagh, Afshin A. Divani

**Affiliations:** 1 Department of Neurology, University of Tennessee Health Science Center, Memphis, TN 38163, USA; 2 Neurological Service, SS Annunziata Hospital, Sulmona, 67039 L’Aquila, Italy; 3 Department of Neurology, University of Mississippi Medical Center, Jackson, MS 39216, USA; 4 Department of Neurology, University of New Mexico, Albuquerque, NM 87131, USA; 5 Department of Neurology, The Ohio State University Wexner Medical Center, Columbus, OH 43210, USA; 6 Department of Neurology, University of North Carolina, Chapel Hill, NC 27599, USA; 7 Department of Advanced Medical and Surgical Sciences, University of Campania, 80138 Naples, Italy; 8 Department of Human Genetics, Punjabi University, Patiala 147002, India; 9 Unidad de Cuidados Intensivos, Sanatorio de la Trinidad San Isidro, Buenos Aires 1640, Argentina; 10 Stroke Division, Department of Neurology, Beth Israel Deaconess Medical Center, Harvard Medical School, Boston, MA 02115, USA

**Keywords:** aneurysmal subarachnoid hemorrhage, vasospasm, delayed cerebral ischemia, early brain injury, spreading depolarization

## Abstract

*Background:* Delayed cerebral ischemia (DCI) is a common and serious complication of aneurysmal subarachnoid hemorrhage (aSAH). Though many clinical trials have looked at therapies for DCI and vasospasm in aSAH, along with reducing rebleeding risks, none have led to improving outcomes in this patient population. We present an up-to-date review of the pathophysiology of DCI and its association with early brain injury (EBI). *Recent Findings***:** Recent studies have demonstrated that EBI, as opposed to delayed brain injury, is the main contributor to downstream pathophysiological mechanisms that play a role in the development of DCI. New predictive models, including advanced monitoring and neuroimaging techniques, can help detect EBI and improve the clinical management of aSAH patients. *Summary:* EBI, the severity of subarachnoid hemorrhage, and physiological/imaging markers can serve as indicators for potential early therapeutics in aSAH. The microcellular milieu and hemodynamic pathomechanisms should remain a focus of researchers and clinicians. With the advancement in understanding the pathophysiology of DCI, we are hopeful that we will make strides toward better outcomes for this unique patient population.

## 1. Introduction

**Figure 1:**
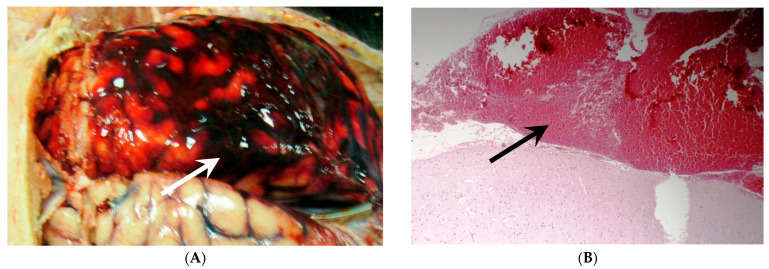
Subarachnoid hemorrhage. The arrows indicate both macroscopic (**A**) and microscopic (**B**) large bleeding (H&E × 10).

Aneurysmal subarachnoid hemorrhage (aSAH) (Figure 1) is a devastating stroke subtype with high morbidity and mortality, accounting for approximately 5% of all strokes. A systematic review of population-based studies showed that the crude incidence of aSAH in 2010 in North America was 6.9 per 100,000 person-years with an annual decline of 0.7% since 1955 [1]. However, despite significant advances in diagnosis and management of aSAH, the overall mortality remains high at 35% (range 20–67%) [2,3] and approximately 20% of survivors experience significant morbidity [4], causing a substantial economic and social burden on the patients, families, and healthcare systems [5]. Delayed cerebral ischemia (DCI) is a feared complication of aSAH, increasing the risk of morbidity and mortality, specifically in patients with a severe clinical presentation at ictus and high SAH burden [6,7]. Published data suggests DCI affects approximately 20–40% of aSAH patients and is an independent poor prognostic factor in this population [8,9,10]. DCI is classically defined as a development of a new neurological deficit(s), impaired consciousness, or infarct on imaging that commences 3 to 4 days after the initial insult and peaks around 7 to 8 days post-bleed [11]. An earlier onset of DCI prior to day 7 has been associated with higher mortality and greater infarct load [12]. Several treatment modalities targeting different pathophysiological pathways causing DCI have been studied. However, there has been minimal change in outcomes due to either DCI prevention or treatment.

The definition and pathophysiology of this complex and multifactorial process have been debated and studied extensively. In 2007, a study with over 3500 patients [13] evaluated independent variables associated with unfavorable outcomes in aSAH. Authors defined the term “symptomatic vasospasm” as a ≥2 point decrease in the Glasgow Coma Scale (GCS) or a ≥2 point increase in the motor score of the National Institute of Health Stroke Scale (NIHSS) lasting >8 h, which has since fallen out of favor due to the lack of clear correlation with outcomes. Interestingly, both “symptomatic vasospasm” and cerebral infarction noted on computer tomography (CT) post-operatively showed a significant association with poor functional outcomes at 3 months, with cerebral infarction showing the highest association with unfavorable outcomes (OR = 5.38, *p* < 0.0001) [13]. In 2010, Vergouwen et al. [14] developed a uniform definition of DCI, incorporating clinical deterioration and cerebral infarction as important DCI characteristics that have been well adopted since (see Table 1). Following the definition standardization, in an observational study from Finland that included 340 aSAH patients, 37.1% fulfilled the consensus definition of clinical deterioration secondary to DCI and were strongly associated with poor outcomes at discharge (OR = 2.65, *p* < 0.001) [15]. This has provided an objective, homogenous, and reliable definition of DCI.

For years, large vessel vasospasm was the recognized complication of DCI, leading to the assumption that vasospasm prevention and treatment were paramount in managing aSAH after securing the ruptured aneurysm. However, clazosentan, which was found to treat and reverse vasospasm successfully, failed to improve outcomes. This is possibly because cerebral infarction, secondary to DCI, often occurs in vascular territories outside of the vasoconstricted territories [8,16,17,18]. Furthermore, DCI has been reported among patients with none or only mild angiographic evidence of vasospasm. Though cerebral vasospasm has been found to occur in 50–70% of aSAH patients, only 20–40% develop DCI [8,13]. Because of this, efforts in the last two decades have been placed on establishing and understanding the pathophysiology of DCI. Hence, the focus has shifted to early brain injury (EBI) and its association with autoregulatory failure, neuroinflammation, and eventually delayed injury (also defined as DCI), with the thought that the treatment of large vessel vasospasms alone might be reactionary and does not address the inciting mechanisms of injury. This review will discuss the current knowledge of pathophysiological mechanisms surrounding EBI and its association with DCI and clinical outcomes after aSAH. In addition, we will discuss the future directions of clinical practice and research to accurately identify various risk factors and strategize therapeutic strategies to improve outcomes in this population.

## 2. Risk Factors Associated with aSAH and DCI

DCI entails modifiable and non-modifiable pre-morbid risk factors. Though a comprehensive discussion of pre-morbid risk factors is outside this review’s scope, Ya et al. [19] noted that smoking was a modifiable risk factor for DCI after aSAH in a meta-analysis. The exact mechanism of this correlation is not well understood, and the role of nicotine is unclear.

Other modifiable and non-modifiable risk factors have been identified in multiple studies. A large multicenter cohort of over 500 patients with aSAH found that female sex, pre-morbid diabetes mellitus, and poor grade aSAH were independent risk factors for DCI [20]. Conversely, Crobeddu et al. [21] reported that older patients (>68 years) with good clinical and radiographic grades were less likely to develop DCI.

Genetic studies have described the role of inheritance patterns in intracranial aneurysm cases, ensuing rupture, and the development of post-rupture complications [22,23]. It has been observed that an individual with a family history of aSAH has a 10% chance of aSAH [24]. A large twins cohort study revealed that the estimated heritability of aSAH is 41% suggesting substantial genetic involvement in its pathogenesis [25]. Candidate gene association studies, meta-analysis, genome-wide association studies (GWAS), and epigenome-wide association studies (EWAS) have exposed several risk variants for unruptured and post-rupture of intracranial aneurysms [26,27]. Interestingly, most (if not all) of the genetic variants observed are unrelated in different studies with discordant inferences. For instance, three meta-analyses have been conducted so far to identify genetic variants responsible for cerebral vasospasm and DCI post-aSAH. The first meta-analysis [28] comprising 16 studies and four genes (endothelial nitric oxide synthase [eNOS], apolipoprotein E [APOE], haptoglobin [Hp], and ryanodine-1 [RYY-1]) demonstrated that carriers of Hp2-2 genotype are at higher risk of developing cerebral vasospasm and DCI. In contrast, another meta-analysis [29] has refuted this claim and showed that Hp2-2 was not associated with either cerebral vasospasm or DCI. The third meta-analysis has suggested that Hp1-2 and Hp1-1 subtypes confer protective effects for cerebral vasospasm and DCI [30]. The possible reasons for such inconsistent results are different sample sizes of the studies deriving variable statistical power, lack of true source of heterogeneity, presence of population stratification, unforeseen epistasis, and miscalculating gene-environmental interactions [31]. A GWAS examining the gene expression array of blood cells post-aSAH has revealed that the angiogenic and pro-inflammatory gene, neuregulin 1 (NRG1), expresses in patients with DCI and predicts DCI with excellent statistics (AUROC: 0.96) [32]. An EWAS on methylation profiles of patients with DCI has revealed that the hypermethylation of two genes (INSR, CDHR5) at cg00441765 and cg11464053 sites is significantly associated with reduced mRNA expression [33]. Another study has examined DNA methylation vis-à-vis cerebral vasospasm and DCI along with an unfavorable Glasgow Outcome Scale (1–3). The results revealed that methylation site cg25713625 of the STEAP3 gene is significantly associated with unfavorable outcomes and can be used as a therapeutic target for the clinical management of cerebral vasospasm and DCI post-aSAH [34].

Genetic studies are used in clinical practice for decision-making and preventing the worst outcomes of complex diseases [35]. Several genetic variants interact with modifiable risk factors to exhibit heterogeneous effects in complex pathologies such as cerebral vasospasm and DCI. Developing polygenic risk scores (PRS) has ignited a ray of hope that individuals can be risk-stratified on their personalized propensity of the disease [36,37]. A study has utilized the effect estimates for generating weighted PRS (wPRS) by involving 125 intracranial aneurysms and 222 acute ischemic stroke patients in a validation study with 296 matched controls [38]. The risk scoring included 29 GWAS (*p* < 5 × 10^−8^) and two already investigated genetic variants (*p* < 0.01), which have shown excellent predictability for intracranial aneurysms (AUROC: 0.95, 95%CI: 0.93–0.97) and acute ischemic stroke (AUROC: 0.842, 95%CI: 0.81–0.88) with a wPRS_IA_ demonstrating a clinical sensitivity of 0.73, specificity of 0.96, and predictability of 0.93 after stratification. This model was able to discriminate intracranial aneurysm patients from controls with an accuracy of 85.5% [38]. Similar future studies will help formulate a quantifiable risk score to improve the clinical decision-making for the prognosis, diagnosis, and therapeutic management in aSAH with formidable complications of cerebral vasospasm and DCI.

## 3. Pathophysiologic and Hemodynamic Factors Associated with aSAH

Arterial blood enters the subarachnoid space, cisterns, and often into the ventricular system at the time of initial aneurysm rupture (ictus), leading to a higher-pressure system. As the intracranial pressure (ICP) rises, the cerebral perfusion pressure (CPP) is decreased (CPP = MAP–ICP). In a normal physiological condition, a drop in CPP can sustain cerebral blood flow (CBF) to a limited extent prior to autoregulatory dysfunction and failure. However, in a sudden and large increase in ICP, such as in high-grade aSAH, CBF is markedly decreased, leading to the vasodilation of distal cerebral arterioles with the concomitant rise in arterial blood pressure that causes an increase in CBF, further increasing the ICP, until a complete cessation of CBF occurs. This leads to a phenomenon known as “transient global cerebral ischemia” [39]. Early or ictal infarcts can occur during the initial insult where early MRI may be helpful to differentiate this from DCI [11]. Weimer et al. [40] compared the whole brain apparent diffusion coefficient (ADC) on MRI in aSAH patients with controls to quantify the degree of early cytotoxic and vasogenic edema and observed significantly higher ADC values in the aSAH group, providing evidence of early brain edema as a possible mechanism for EBI.

The drop in CPP due to elevated ICP described at ictus is followed closely by a sympathetic catecholamine surge. Injury to the hypothalamus and brainstem, particularly the medulla, due to increased pressure leads to the release of catecholamines mediated by the rostral ventrolateral medulla and the paraventricular nucleus in the hypothalamus [41]. In 1963, Crompton [42] described this phenomenon after aSAH as related to necrosis or hemorrhage of the hypothalamus or damage or vasoconstriction of the arteries that feed it. More recently, this effect was well documented using the diffusion tensor imaging tractography technique showing a decrement in fractional anisotropy and an increase in ADC [43]. The injuries associated with the initial rise in ICP are thought to lead to the accumulation of carbon dioxide and glutamate, which gives rise to reactive astrocytes, and sympathetic activation, followed by secondary activation of the renin-angiotensin-aldosterone system in the kidney [44,45,46]. This sympathetic reflex has traditionally been recognized as leading to the well-known Cushing response from sympathetic over-excitation due to elevated ICP. The Cushing reflex occurs during prolonged ICP elevation with Lundberg A or plateau waves [47]. However, there has been evidence of an early Cushing-like response, thought to occur in the sudden and early, rather than sustained, rise in ICP after brain injury; this is associated with an intracranial baroreflex and sympatho-excitatory response to raise systemic blood pressure in an attempt to maintain CPP and CBF [48]. Moreover, the catecholamine surge can also lead to endothelin activation, thought to play a role in vasospasm, as well as cytokine release that leads to hyperglycemia, hypokalemia, and leukocytosis. This pathophysiology is the cause of cardiac, lung, renal, and other systemic complications, with a proven dramatic increase in plasma norepinephrine in the setting of cardiac injury in these patients [44,49]. In addition, the insular cortex stimulation with blood spilled due to SAH is realized in paroxysmal sympathetic hyperactivity syndrome [50]. These processes can cause hypotension and hypoxia, which further potentiates brain injury. Though this process starts at the ictus, it continues past the initial injury, causing the process known as EBI.

The prevention of worsening from initial ischemia is of paramount importance post-aSAH. Management of early complications, like rebleeding and hydrocephalus, is an important first step in managing aSAH. Cortical tissue irritation from subarachnoid blood can induce clinical seizures at the onset, most commonly occurring within the first 24 h. This complication occurs in over a quarter of patients, contributing to further increases in cerebral metabolic demand and a secondary increase in ICP due to cerebral blood flow elevations during the ictal period, potentiating ischemia and causing deleterious effects [51]. To manage cerebral autoregulatory failure, bedside blood pressure management is most commonly used, and the maintenance of mean arterial pressure is a mainstay of aSAH management.

In addition to the aforementioned pathophysiological mechanisms, molecular changes contribute to cerebral edema and DCI development. Macrophage migratory inhibitory factor (MIF), a homotrimer protein, has been demonstrated to function as a proinflammatory cytokine capable of activating inflammatory responses in the central nervous system [52,53]. In experimental studies, MIF is released from activated glial cells, which further activate the release of inflammatory mediators from astrocytes leading to brain injury by promoting neuronal cell death [54,55,56]. In recent studies, serum MIF concentrations have been shown to be associated with severity and long-term outcomes in patients with acute ischemic stroke, traumatic brain injury, and spontaneous intracerebral hemorrhage [57,58,59]. Similarly, MIF has also been studied as a marker of the severity of brain injury in patients with aSAH. In a prospective study by Chen et al. [53], serum MIF concentrations were found to be significantly higher in patients with aSAH as compared to controls (30.1 ng/mL vs. 7.9 ng/mL; *p* < 0.001). After adjusting for confounding variables (World Federation of Neurosurgical Societies [WFNS] and Fisher scores), serum MIF was identified as an independent predictor for 6-month unfavorable outcomes (Glasgow Outcome Scale Extended, 1–4) in aSAH patients [53]. In another study by Yang et al. [60], higher serum MIF levels were seen in patients who developed DCI as compared to patients without DCI (26.4 [IQR: 22.6–32.4] ng/mL vs. 20.4 [IQR:16.4–24.6] ng/mL, *p* < 0.001). Furthermore, MIF showed a greater discriminatory ability for DCI than the APACHE-II score, C-reactive protein, and interleukin 6 (IL-6) with an AUC of 0.780 (95% CI, 0.710–0.849).

**Figure 2:**
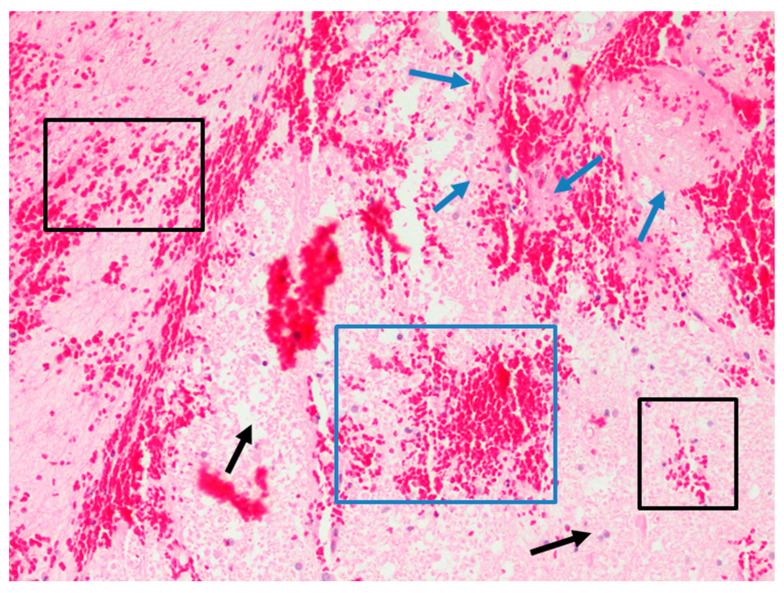
H&E section (×20) showing distribution pattern of erythrocyte extravasations in cerebral ischemia. Intraparenchymal zone in which erythrocyte extravasations are partly diffused (black box), partly wider (blue box), in a context of ischemic necrosis (blue arrow) and edema with aspects of hypoxic cell damage (black arrow).

Upregulation of vascular endothelial growth factor and aquaporin-4, increased levels of bradykinin, cytokine release of IL-6, and matrix metalloproteinase-9 are possible targets for treatment, although studies have shown mixed results [51,61]. Drier et al. [62] described that spreading depolarization in the gray matter led to cytotoxic edema, which resulted in ischemia, shown microscopically in Figure 2, leading to neuronal cell death and DCI. Predictive models and prognostication of early edema in aSAH are vast research topics. Wartenberg et al. [63] studied 21 high-grade aSAH patients with early MRI, noting that early ADC diffusion restriction correlates in regions not seen on initial CT. Of those patients, 71% died, and only 12% had a satisfactory outcome. This study proposed that infarcts on CT imaging provided a small picture, and that early MRI could help in noticing and managing early ischemia. In 2018, Ahn et al. [64] evaluated the presence of early edema and noted it as a possible surrogate for EBI and predictor of DCI by designing the Subarachnoid Hemorrhage Early Brain Edema Score (SEBES) that has been recognized as an independent predictor of DCI and is described later in this review. Aside from survival and functional outcome, cerebral edema has also been noted as a predictor of poor cognitive outcomes [61].

### Early Brain Injury

EBI is believed to occur in the first 72 h following ictus and is a precursor for DCI. Impaired autoregulation is also most prominent in the first 72 h, associated with the increased risk of DCI [65]. The catecholamine surge at ictus continues up to 7 to 10 days post-rupture [44]. As previously mentioned, glutamate accumulation and impaired uptake are part of this process. This causes NMDA receptor activation leading to calcium ion flooding the cell, inducing apoptosis and cell necrosis, as well as the breakdown of the blood-brain barrier (BBB), which all contribute to EBI [65].

Endothelial dysfunction is thought to be a major contributor to vasoconstriction. Oxy- and deoxyhemoglobin damage healthy neurons that secrete neuronal nitric oxide synthase in the brain vessels and parenchyma. The decrease in nitric oxide level needed for vasodilation leads to vasoconstriction. The normal reactionary increase in endothelial nitric oxide synthase is also impaired, which causes continued vasoconstriction until nitric oxide is restored to a normal level [65]. This is also implicated in BBB breakdown due to associated apoptosis and increased permeability, especially in microvessels [65]. Activation of calcium channels and enhanced membrane depolarization are thought to lead to further microvessel spasms [66].

Platelet aggregation is another important contributor to EBI. In animal models, microthrombi from platelet aggregates have been noted as early as 10 min following aSAH. Punctate infarcts on MRI could be associated with microthrombi and have been shown to predict worse outcomes [67]. Another study that evaluated MRI diffusion restriction in the first four days after poor grade aSAH demonstrated that 86% of patients had diffusion restriction and ADC correlates that were not seen on CT [63].

Cellular loss of ionic gradient leads to cortical spreading depolarization, which is seen as a negative direct current, noting differences in depolarization. Sodium and water enter the depolarized cell, leading to cellular swelling and necrosis. This sustained neuronal depolarization presents as slow-moving and propagating waves, detected using subdural electrodes. In severe cases, this can lead to spreading depolarization-induced spreading depression, which is a decline in the amplitude of the alternating current, or loss of spontaneous electrical activity in the brain [62]. This is associated with injury in a bimodal fashion, with both early and delayed brain injury after aSAH [17,51]. In a recently published pilot clinical trial on depolarizations in ischemia after subarachnoid hemorrhage (DISCHARGE-1) [68], the primary outcome was the peak total depression time from spreading depolarization that was indicative of DCI. The authors hypothesized that a 60-min cutoff of spreading depolarization-induced depression would predict delayed infarction with high sensitivity (>0.6) and specificity (>0.8). However, the study’s results revealed that this time interval was too short of reaching the desired specificity of 0.80 (specificity was 0.59), though it reached higher than desired sensitivity (0.76). A 180-min cutoff was found to have lower sensitivity (0.62), but higher specificity (0.83). However, the 60-min cutoff had a higher sensitivity and specificity for predicting DCI than the defined cerebral infarction. In their conclusion, Dreier et al. [68] found that spreading depolarizations were likely an independent marker of brain injury post-aSAH. One episode of spreading depolarization alone was shown to significantly affect the risk of poor outcomes in their studied population. In another study of 19 subjects, Owen et al. [69] reviewed cerebral autoregulation and spreading depolarizations simultaneously through multimodal monitoring. Spreading depolarization was associated with impaired cerebral autoregulation, proposing a negative feedback phenomenon from impaired cerebral autoregulation that leads to decreased CBF and spreading depolarization, leading back to further worsening of cerebral autoregulation. Though there is no consensus, given these findings, it is plausible that cortical spreading depression provokes ischemia. These important studies further support the use of advanced neuromonitoring, especially in high-grade aSAH that also has the potential to provide us with a therapeutic target. Carlson et al. [70] published a pilot study of ketamine to suppress spreading depolarizations in traumatic brain injury and aSAH patients, with a subsequent study to look at an alternative N-methyl-D-aspartate receptor antagonist, memantine, which seems to be a promising alternative [71].

## 4. Recent Developments

### 4.1. Subarachnoid Hemorrhage Early Brain Edema Score (SEBES)

**Figure 3:**
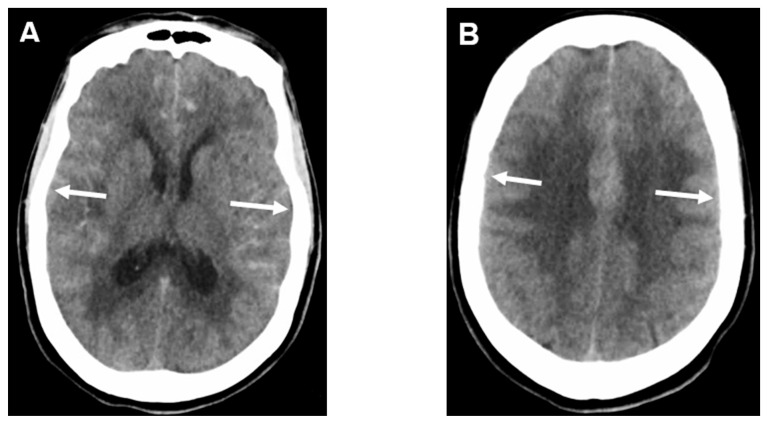
SEBES score calculation from non-contrast CT of the head. (**A**) Slice 1 with 2 points for bilateral effacement of sulci. (**B**) Slice 2 of the same CT with 2 points for bilateral sulcal effacement. The total SEBES score for this scan is 4.

The SEBES score is a semi-quantitative CT grading scale that was developed as a novel radiographic marker of EBI, also helpful in predicting DCI and unfavorable outcomes. The score is based on a scale of 0–4 (where 4 presents the worst) and calculated similarly to the Alberta Stroke Program Early CT Score (ASPECTS) [72] in acute ischemic stroke, at two separate slices: Slice 1: the level of the insular cortex, showing the thalamus and basal ganglia, and Slice 2: the level of the centrum semiovale above the level of the lateral ventricle. A point is assigned for each side with effacement of the sulci, at each level, as seen in Figure 3. In a prospective study that included 164 patients, high-grade Hunt and Hess (HH) and higher WFNS grade were identified as predictors of high-grade SEBES score (defined as a score of 3 or 4). After adjusting for covariates, SEBES was identified as an independent predictor of DCI (OR = 2.24, 95% CI: 1.58–3.17) and unfavorable outcome (OR = 3.45, 95% CI: 1.95–6.07) [64]. In another study by Said et al. [73], independent associations were found between higher SEBES scores (3 or 4) and the need for medical management of ICP, decompressive craniectomy, development of cerebral infarction, as well as unfavorable outcomes. In an observational cohort, Rass et al. [74] used the SEBES score repeatedly to follow the course of edema. They concluded that the score could identify patients with delayed resolution of early brain edema. As stated, aggressive control of cerebral edema, and the factors that play a role in the late resolution, should be part of the initial management of these patients using the SEBES score.

### 4.2. Volumetric Analysis of Edema

Dhar and his team have recently studied volumetric analysis of global cerebral edema (GCE) as a predictor of poor outcomes after aSAH [75], where the selective sulcal volume (SSV) and the CSF volume above the lateral ventricles were measured using an automated machine learning algorithm and studied as a quantitative biomarker of GCE. In this study, an SSV cutoff below 5 mL accurately identified GCE in patients with aSAH. Furthermore, early SSV (i.e., the lowest SSV within 72 h of ictus) was noted to be an independent predictor of poor outcomes with the strongest impact on the prediction of GCE in younger patients (<70 years old).

### 4.3. Multimodal Monitoring, Predictive Models, and Diagnosis of Dci

Multimodal monitoring (Table 2) is becoming more widely used in this patient population. It has been shown to aid in detecting DCI, especially in patients with limited reliable secondary brain injury examination [76,77]. Lazaridis’s summary of cerebral shock explains different pathophysiologic profiles associated with these monitoring techniques, summarized in Table 3 [78]. Brain tissue oxygen (PbtO2) and cerebral microdialysis can be used for individualized patient care vis-à-vis the above mechanisms of injury from local and regional hypoxia. Trend analysis of PbtO2 helps distinguish early from delayed injury, where a drop in PbtO2 has been shown to correlate with vasospasm and DCI [10]. Cerebral microdialysis can give data on the brain’s metabolic profile that can be affected days before the irreversible injury [10]. The lactate-to-pyruvate ratio (LPR) has been studied as a potential biomarker in aSAH and other neurologic injuries. An increase in the LPR can be associated with ischemia and poor patient outcomes [79]. A recent study [80] used a multimodal approach to create a useful diagnostic tool to detect patients at risk for ischemia. A fraction of inspired oxygen (FiO2) challenge was completed that would normally increase the PbtO2. The investigators noted that patients with higher cerebral lactate had less increase in PbtO2 and concluded that a less-than-expected increase in PbtO2 vis-à-vis FiO2 challenge could be a surrogate for elevated cerebral lactate and risk for ischemia. Megjhani et al. [81] measured CPP in association with PbtO2. A non-linear relationship was found, but it showed that hypoperfusion and less than optimal values of CPP were associated with decreased PbtO2 and regional brain hypoxia. Because of a nonstandard approach related to these modalities, studies of invasive multimodal monitoring have produced mixed results. The ability to interpret results, recognize, and treat patients based on those results are user and training-dependent. However, these tools demonstrate the focus on microcellular milieu data, contrasted with the previous thought of management based on large vessel vasospasm alone.

Near-infrared spectroscopy (NIRS) is a non-invasive brain tissue oxygenation monitoring device, but its use is limited due to the ability to monitor mainly the frontal lobe. De Courson et al. looked at the correlation between PbtO2 and NIRS measurements among 51 SAH patients and found no correlation [82]. Pupillometry has been well studied as a tool for early detection of potential worsening in brain injury cases. Regarding aSAH, Aoun et al. [83] looked at 56 patients with transcranial doppler and Neurological Pupil index (NPi) measurements in combination. In this study, sonographic vasospasm was not significantly associated with the NPi measurement, but sonographic vasospasm and abnormal decreases in NPi were each associated with DCI.

Cortical spreading depolarization and depression can be monitored along with continuous electroencephalogram (cEEG) to detect changes such as decreasing relative alpha variability, decreasing alpha-delta ratio, worsening focal slowing, or late-appearing epileptiform abnormalities that are considered EEG alarms [84,85,86]. This approach [85,86] could predict DCI days before the actual event, which is an exciting advancement and also shows this as a dynamic pathophysiologic process.

Vasospasm does occur around the period of DCI and cerebral infarction, though not always found to correlate with location or degree of ischemia. Transcranial Doppler is a non-invasive tool that can detect possible vasospasm. Mean flow velocities in the middle and anterior cerebral arteries are typically considered abnormal for >120 cm/s, with >200 cm/s consistent with severe vasospasm [10]. CT angiogram is another valuable non-invasive imaging modality that can be used for diagnosing cerebral vasospasm. However, catheter-based angiography remains the gold standard, giving both the ability to diagnose and treat. CT perfusion (CTP) imaging has been studied as a potential tool in detecting DCI. In a systematic review that included 345 patients, aSAH patients with perfusion deficits on CTP imaging demonstrated an approximately 23-fold higher likelihood of developing DCI than patients with normal CTP results [87]. Further, a mean transit time (MTT) exceeding 6.4 s, or regional CBF below 25 mL/100 g/min correlated with the development of DCI in the study [88]. In another study by Cremers et al. [89], aSAH patients with assessed perfusion deficit with CTP at the time of clinical deterioration were more often noted to develop infarction on follow-up imaging (88%) as compared with patients without a perfusion deficit (38%). CTP imaging can provide detailed information on microvascular circulation. Therefore, its combination with CT angiography or digital subtraction angiography can provide a complete view of cerebral circulation [90,91,92,93]. However, due to high variability in post-processing methods and equipment used, along with inconsistency with quantitative variables and their thresholds for detecting DCI, the question of the sensitivity of CTP is a question of clinicians [94]. Further prospective studies are required before the wide acceptance of CTP as a promising tool in detecting DCI.

## 5. Future Directions

### Clinical and Risk Factor Assessment

Several studies have resulted in different grading systems based on clinical or radiological factors to predict DCI or outcomes to guide treatment [95]. The GCS, HH, and WFNS are the widely used clinical grading scales, focusing on signs and symptoms; however, these grading structures do not take into account the quantity and severity of bleeding. With the improved CT techniques, some radiographic scales have been further developed. Quantifying thickness and area of subarachnoid blood on CT scans, the Fisher Scale and the modified Fisher Scale can predict the incidence of cerebral vasospasm and DCI [7]. The SEBES is a new scoring tool that displays the degree of EBI to predict the incidence of DCI. However, further research is needed to determine its accuracy and effectiveness [64]. The main issue with radiological scales is the absence of clinical symptoms. Thus, a few combined grading systems have been promoted, including VASOGRADE (VG) [96] and the HAIR [97] scales described in Table 4, to predict DCI and in-hospital mortality respectively [97]. However, those grading structures do not consider the importance of EBI. New studies have recognized that the incidence of DCI is associated with the severity of EBI after SAH [98]. Other scores include combining multiple variables of admission data [99] and more dynamic scores to give clinicians insight into the possibility of current DCI [100].

Multiple predictive models have been recommended for DCI. Coated platelets, a subset of platelet that keeps the prothrombotic substance attached to the platelet and a marker for thrombogenicity, was studied to look at DCI prediction. The trend of coated platelets after initial ictus was found to be a reliable predictor of DCI [101]. Machine-learning capabilities for better prediction have been shown to be beneficial in multiple studies and are promising roads for future research [102,103].

## 6. Necessity for Ongoing Research

Further reliable predictive models and markers to determine patients at high risk for DCI and other complications following aSAH are an important continued endeavor. As mentioned previously regarding genetic predisposition to DCI, the potential of polymorphisms associated with nitric oxide synthase (NOS), specifically the neuronal type, has shown promising bench research results. Endothelial NOS was not significantly shown to increase DCI or vasospasm risk in a meta-analysis [28]. Although challenging due to the unavailability of either pre- or post-morbid genetic testing, further prospective studies may shed a better light on the role of endothelial NOS.

The oxidative stress associated with the initial catecholamine surge at the ictus has been studied vis-à-vis possible mechanisms to address the complications arising from that excitatory response. Renal denervation was investigated to stabilize the sympathetic response by deactivating efferent sympathetic nerves in the periphery. This reduced angiotensin-II and endothelin-1 in animal models and offered hope for inhibiting vasoconstriction and vasospasm [45]. Although previous studies have proven that treatment of vasospasm may not improve outcomes, the idea of continued research to address the ictal sympathetic surge is possible.

A multicenter study with a large sample size and standardized variables is needed regarding aSAH to find the best generalizable and reproducible results to lead to a change in clinical practice. Using relevant preclinical models is an essential part of conducting translational studies [104]. This is necessary to address the limited treatment options that currently exist for aSAH and DCI with a significant degree of poor outcomes.

## 7. Conclusions

The primary goal of neurointensivists in clinical practice is to ameliorate the burden of morbidity and mortality associated with aSAH. DCI is a complex multifactorial pathophysiological process that starts early post-aSAH, with risk factors present before and at ictus. The pathophysiology of EBI and the mechanisms mentioned earlier act as a substrate for the development of DCI. This includes the well-known mechanism of vasoconstriction and spasm, focusing on the sympathetic surge and cytokine release, microthrombosis, and BBB breakdown. Early focus on EBI and global cerebral edema are essential in managing aSAH. Our research goals should continue to change and attempt to address these newer pathomechanisms in a search for improved outcomes that can be translated into routine clinical practice.

## Figures and Tables

**Table 1:** Consensus definition of clinical deterioration and cerebral infarction due to DCI.

**Clinical Deterioration Due to DCI**	Occurrence of focal neurological impairment (such as hemiparesis, aphasia, apraxia, hemianopia, or neglect), or a decrease of at least 2 points on the GCS (either on the total score or on one of its individual components [eye, motor on either side, verbal]). This should last for at least 1 h, is not apparent immediately after aneurysm occlusion, and cannot be attributed to other causes by means of clinical assessment, CT or MRI scanning of the brain, and appropriate laboratory studies.
**Cerebral Infarction Due to DCI**	Presence of cerebral infarction on CT or MR scan of the brain within 6 weeks after SAH, or on the latest CT or MR scan made before death within 6 weeks, or proven at autopsy, not present on the CT or MR scan between 24 and 48 h after early aneurysm occlusion, and not attributable to other causes such as surgical clipping or endovascular treatment. Hypodensities on CT imaging resulting from ventricular catheter or intraparenchymal hematoma should not be regarded as cerebral infarctions from DCI.

**Table 2:** Multimodal Advanced Neuromonitoring.

Neurophysiological Parameters	Subtypes and Notes
**Cerebral Autoregulation**	Relies on multiple modalities to maintain preferred CPP Helpful to have an arterial line and advanced hemodynamic monitoring system
**Cerebral Blood Flow**	Transcranial Doppler Thermal Diffusion Flowmetry (Thermal Clearance Method) CT or MR perfusion Positron emission tomography—rarely used in practice
**Cerebral Oxygenation**	PbtO2 NIRS Jugular venous oximetry
**Intracranial Pressure**	External ventricular drain—benefit of draining CSF as well as monitoring ICP Parenchymal probe Subdural ICP monitor
**Electroencephalography (EEG)**	Conventional scalp EEG—non-invasive technique, easy to remove and replace Subdural or depth electrodes
**Pupillometry**	Abnormal decreases in NPi may be an early sign of DCI
**Cerebral Microdialysis**	Clinical utility unclear

**Table 3:** Pathophysiologic Types of Cerebral Shock (adapted from Brain Shock-Toward Pathophysiologic Phenotyping in Traumatic Brain Injury [78]). Brain tissue oxygen (PbtO2); lactate/pyruvate ratio (LPR); cerebral blood flow (CBF); cerebral perfusion pressure (CPP); intracranial pressure (ICP); cortical spreading depression (CSD). If a neuromonitoring result is not listed, it is either unchanged or unpredictable.

Pathophysiologic Type	Neuromonitoring Result	Underlying Pathophysiology and Management
Flow Dependent	↓: PbtO2, glucose, pyruvate ↑: lactate, LPR	Suboptimal CBF → optimize hemodynamic parameters and CPP
Flow Independent Oxygen Diffusion Limitation	↓: PbtO2 ↑: lactate, LPR	Intracellular/interstitial edema → appropriately manage cerebral edema
Flow Independent Energy Production (Mitochondrial) Failure	↓: lactate, LPR, possibly pyruvate ↑: glucose	Management is unclear
Flow Independent Microvascular Shunting	↓: PbtO2 (from ↑CBF) ↑: glucose, lactate	Microvascular shunting → appropriately manage ICP
Low Extraction	↓: PbtO2, pyruvate ↑: lactate, LPR	Hypoxemic, anemic or high-affinity hypoxia → treat appropriate underlying cause to improve oxygenation
Hypermetabolic	↓: PbtO2, glucose, pyruvate ↑: lactate, LPR Similar profile to flow dependent	Increase in metabolic demand → Avoid hyperthermia, seizures, CSD; consider sedation if appropriate

**Table 4:** VASOGRADE and HAIR scores, from respective studies, for aneurysmal subarachnoid hemorrhage. Modified Fisher scale (mF), World Federation of Neurosurgical Societies scale (WFNS); Hunt and Hess score (HH); Intraventricular hemorrhage (IVH).

Score	Purpose	Score components	Findings
VASOGRADE (N = 746)	DCI risk stratification	Green: mF 1–2 AND WFNS 1–2 Yellow: mF 3–4 AND WFNS 1–3 Red: WFNS 4–5 regardless of mF grade	Yellow: tendency to DCI compared to Green Red: 3-fold increased risk of DCI compared to Green
HAIR (N = 400–score development) (N = 302 –score validation)	In-hospital mortality risk stratification	Score 0–8 total HH: 1–3 = 0 pts; 4 = 1 pt; 5 = 4 pts Age: <60 = 0 pts; 60–80 = 1 pt; ≥80 = 2 pts IVH: No = 0 pts; Yes = 1 pt Re-bleed (within 24hrs): No = 0 pts; Yes = 1 pt	Increase in HAIR score increase in rate of in-hospital mortality

## Data Availability

Data sharing not applicable.

## Abbreviations

aSAH	Aneurysmal subarachnoid hemorrhage
ADC	Apparent diffusion coefficient
APOE	Apolipoprotein E
CBF	Cerebral blood flow
CPP	Cerebral perfusion pressure
CT	Computed tomography
DCI	Delayed cerebral ischemia
EBI	Early brain injury
EEG	Electroencephalogram
eNOS	Endothelial nitric oxide synthase
EWAS	Epigenome wide association studies
FiO2	Fraction of inspired oxygen
GCS	Glasgow Coma Scale
GWAS	genome wide association studies
Hp	Haptoglobin
ICP	Intracranial pressure
IL-6	Interleukin 6
MAP	Mean arterial pressure
MIF	Macrophage migratory inhibitory factor
MRI	Magnetic resonance imaging
NIHSS	National Institute of Health Stroke Scale
NIRS	Near-infrared spectroscopy
NOS	Nitric oxide synthase
Npi	Neurological Pupil index
NRG1	Neuregulin 1
OR	Odds ratio
PbtO2	Brain tissue oxygenation
PRS	Polygenic risk scores
RYY-1	Ryanodine-1
SEBES	Subarachnoid hemorrhage early brain edema score
wPRS	Weighted PRS
